# Post-COVID Mucormycosis Involving Mandible: A Rare Phenomenon

**DOI:** 10.7759/cureus.34260

**Published:** 2023-01-27

**Authors:** Manish Raghani, Hafiz Md Nasimuddin Ansari, Abdul Hafeez A, Subham Agarwal

**Affiliations:** 1 Oral and Maxillofacial Surgery, All India Institute of Medical Sciences, Raipur, Raipur, IND

**Keywords:** covid-associated mucormycosis, covid-19 associated mucormycosis, post covid mucormycosis, osteomyelitis, fungal, post-covid infections, mandibular mucormycosis

## Abstract

Mucormycosis is a fungal disease involving predominantly the paranasal sinuses and further spreading to the orbit and cerebral regions. It does rarely affect the pulmonary region and gastrointestinal regions. This disease is seen more in a very serious state, where the tissues undergo necrosis and cause huge morbidity and, in some cases, end up being fatal. The disease was common in individuals with an immune-compromised state, thus more commonly presenting in individuals with uncontrolled diabetes. The disease is usually acquired through coming into contact with spores of the fungus Mucormycetes through the nose, and the fungi invade the paranasal regions, colonize, and spread locally with angio-invasion and relying on host ferritin for survival, thereby causing tissue necrosis. The incidence of mucormycosis had increased considerably post-COVID-19 due to host immune factors. This fungus commonly spreads from paranasal regions to the cranial direction through orbit. The spread is rapid, thus needing early medical and surgical intervention. The spread of infection from the paranasal regions to the caudally placed mandible is very rarely seen. In this paper, we present three cases of mucormycosis spreading caudally and involving the mandibular regions.

## Introduction

Mucormycosis (phycomycosis or zygomycosis) occurs by coming into contact with the spores of some molds called mucormycetes from fungi, namely Mucor species or Rhizopus species. This uncommon disease usually affects the head and neck region but sometimes also affects the lungs, rarely involving the skin and gastrointestinal regions too. In the rhino-cerebral form, mucormycosis usually begins in the nose, spreads to involve the paranasal sinuses and palate, and spreads fast to involve the orbit and central nervous system, sometimes causing death. Mandibular involvement with mucormycosis is rare, and to date, only a few cases have been reported in the literature. In recent times, this opportunistic infection was predominant to occur in patients recovering from COVID-19 infection. This post-COVID-associated mucormycosis has been reported globally; however, in 2021, it reached alarming proportions in India. This angio-invasive disease was found to occur in individuals with various immunosuppressive conditions like uncontrolled diabetes mellitus, corticosteroid therapy, etc. [1,2]. But in a SARS-COVID-19 patient, the interaction of several factors like immune system deregulation, steroid therapy, and exacerbation of pre-existing diabetes may allow the mucoralean fungi to invade the host easily [3]. In this article, we summarize our experience in dealing with a series of three cases of mucormycosis involving the mandible, which were reported to the Department of Dentistry at AIIMS Raipur.

## Case presentation

Case 1

A 47-year-old male reported complaints of intra-oral mucosal ulcerations and a burning mouth for a duration of ten days. The patient reported a history of a lancinating type of pain in the upper right back teeth, aggravated upon chewing food, and often accompanied by a headache. The patient also gave a history of altered sensations in the right infraorbital region and chin region. No history of any active pus discharge was elicited in oro-nasal cavities. The patient gave a brief hospitalization history for SARS-CoV-2 infection and required oxygen therapy, medical treatment with corticosteroids, low molecular weight heparin, and remdesivir. The patient had uncontrolled blood glucose shortly after acquiring the COVID-19 infection and had been on insulin therapy since then. On clinical examination, a 2 cm × 1 cm oval whitish plaque ulcerating lesion was found in the pre-maxillary region with no evidence of oro-nasal communication, and no active pus discharge was noted. Miller’s grade IV dental mobility of the right-side upper posterior teeth was noted with severe gingival recession and exposed necrotic alveolar bone. The patient had poor overall oral hygiene and calculus deposits (+++). Paresthesia of the right anterior cheek was confirmed through a two-point discrimination test. A provisional diagnosis of fungal invasion was made considering the history and brief clinical examination. Magnetic resonance imaging (MRI) of the para-nasal sinus (PNS) region revealed hyper-intense mucosal thickening suggestive of fungating sinusitis in the bilateral maxillary sinus, ethmoid sinus, and right sphenoidal sinus. Immediate medical management was started. A fungal biopsy of the sinus membrane was done using the Caldwell-Luc approach for the potassium hydroxide (KOH) test to isolate fungi. Medical management was started with intravenous (IV) liposomal amphotericin B (LAMB) immediately, and the patient was planned for surgical debridement at the earliest. The liposomal amphotericin B was given at a dosage of 150 mg/day with adequate pre-hydration and post-hydration.

**Figure 1:**
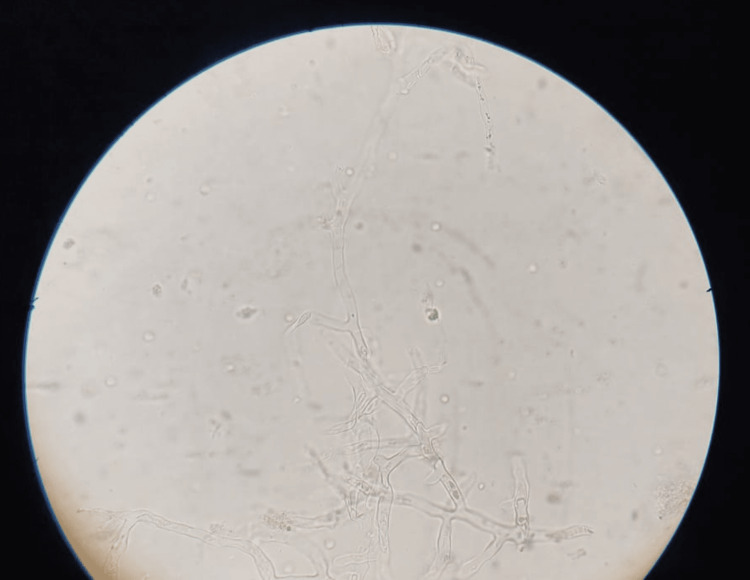
Potassium hydroxide mount showing fungal hyphae elements suggestive of mucormycosis

**Figure 2:**
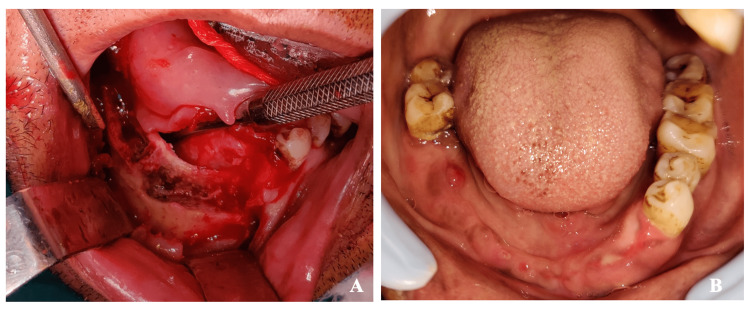
Case 1 - mandibular mucormycosis (A) Intra-operative picture post debridement of sequestrum until bleeding cancellous bone encountered and (B) post-operative follow-up at six months showing no evidence of residual disease and healed ridge.

Within the next two days, the patient complained of persistent pain in the lower front teeth. Erythematous and inflammatory changes involving the lower anterior gingival complex with pre-existing generalized recession were noted. A slowly developing swelling in the chin region about 2 cm × 1 cm in size with tense and well-defined margins was developed in a short span of time. An orthopantomogram (OPG) revealed generalized horizontal bone loss of the lower alveolus without any rarefaction of the corpus of the mandible. The patient was scheduled for dental extraction of the lower anterior teeth and debridement/curettage as a part of the eradication of foci of infection. What started off as trivial dental pain in the lower anterior mandible without any radiographic evidence of pathology was found to be necrosis of the alveolar mandible. The freshly extracted socket had no bleeding whatsoever, which added to the speculation. More intense surgical exploration revealed necrotic cancellous bone with dark gray discoloration and a foul smell, with the bilateral mental nerve being involved and not being preserved. Dental extractions had to be followed by a marginal mandibulectomy to debride the necrotic mass and yield underlying healthy bleeding viable bone. KOH examination of mandibular necrotic bone was positive for broad aseptate hyphae suggestive of mucormycosis (Figure 1). Post-operatively, the patient had mild dehiscence with pus discharge, which was effectively controlled with antimicrobial therapy followed by secondary healing. The anti-fungal therapy was continued until the cumulative dosage of LAMB was set at 2500 mg, followed by oral posaconazole 300 mg for two weeks (Figure 2).

Case 2

**Figure 3:**
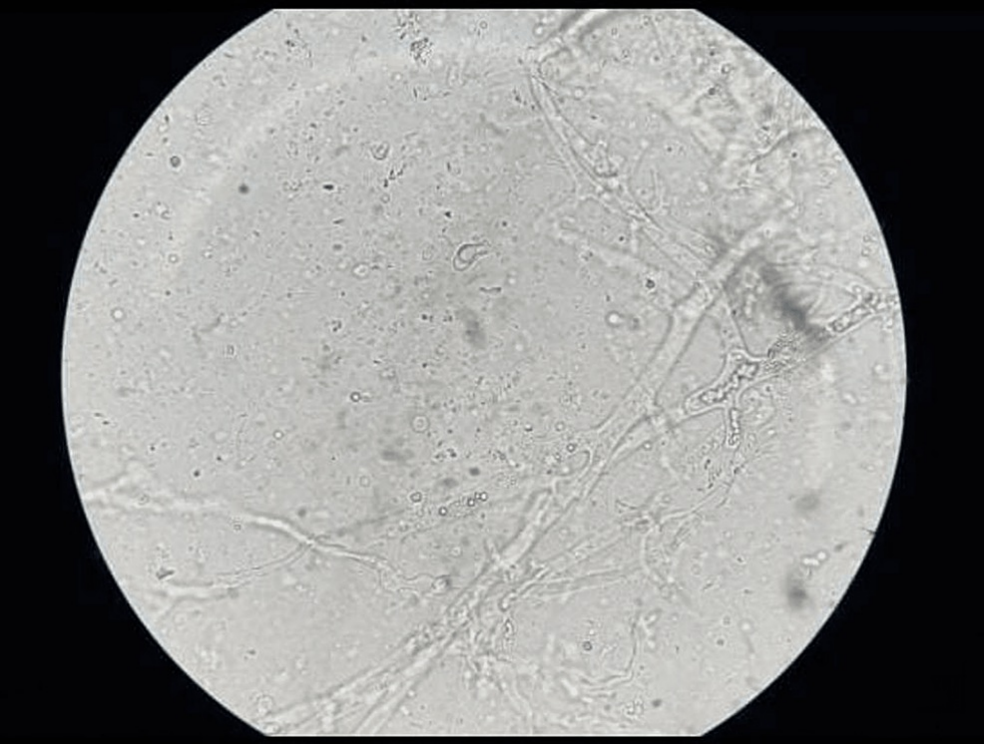
Potassium hydroxide mount picture for mandible of the second case

A 50-year-old male reported pain in the lower right back tooth region radiating to the head over the past month. The patient was briefly hospitalized for SARS-CoV-2 infection for 3 days and was under steroid therapy. The patient gave a history of areca nut chewing for the past two years. The patient is a known diabetic and has been under oral hypoglycemic agents for three years. The patient gave a history of active discharge from gums for the past ten days, accompanied by dull pain. On examination, poor oral hygiene was noted with calculus deposits (+++). Edematous and bogging gingiva with multiple pustules were noted in both the dentate arches. Miller’s grade IV dental mobility was present with gingival recession and exposed necrotic bone. No perforation or oro-nasal communication was noted. Active discharge was noted around the teeth of the left mandibular second premolar region with gingival recession and an altered sensation of the chin and lower lip. The NCCT face revealed osteomyelitis changes in the alveolar process of the bilateral maxilla and mandible (right > left), with cortical breach mainly in relation to the lower incisors and mucosal thickening in the bilateral maxillary sinus and sphenoid sinus. KOH examination of necrotic bone showed broad aseptate hyphae, rendering a definitive diagnosis of both maxillary and mandibular mucormycosis (Figure 3).

**Figure 4:**
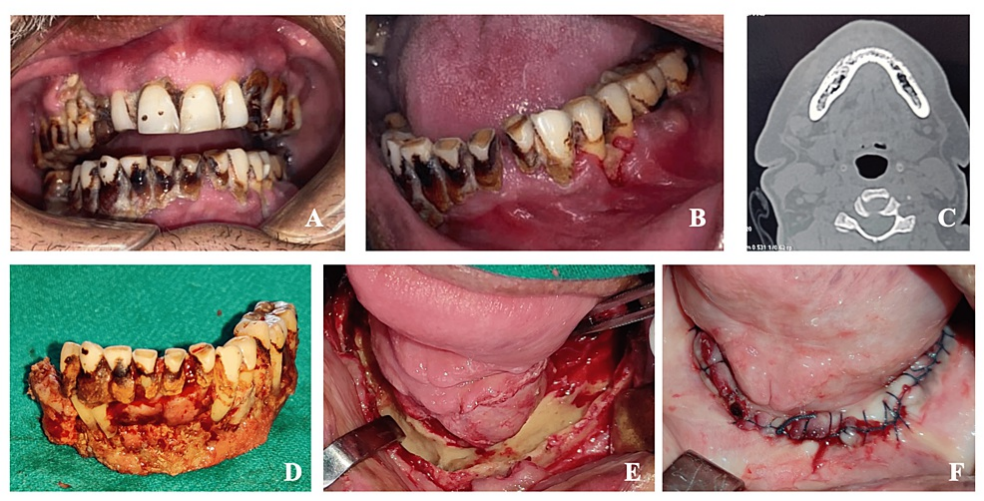
Case 2 - mandibular mucormycosis (A) Preoperative presentation with gross dissemination to rhinomaxillary and mandibular complex, (B) mandibular dentition showing compromised periodontal status, (C) CT-axial section of mandible showing necrotic evidence, (D) resected specimen of necrotic bone, (E) cortical bone preserved and cancellous bone debrided in the lower border of the mandible, and (F) closure of the defect after mucosal advancement.

The patient underwent bilateral subtotal maxillectomy with marginal mandibulectomy. Mandibular cancellous marrow was found to be entirely necrosed with relatively intact cortical bony plates. The bilateral mental nerve was not salvageable. The necrotic sequestrum was completely debrided, preserving the cortical plates (Figure 4). Post-operative wound healing was difficult, with surgical wound dehiscence and active discharge controlled by metronidazole topical gel and amphotericin topical gel, and the patient required secondary local surgical debridement. The patient was also given IV liposomal amphotericin B (150 mg/day) for an extended period until the cumulative dosage reached 2500 mg, and was followed up with oral posaconazole at 300 mg per day.

Case 3

**Figure 5:**
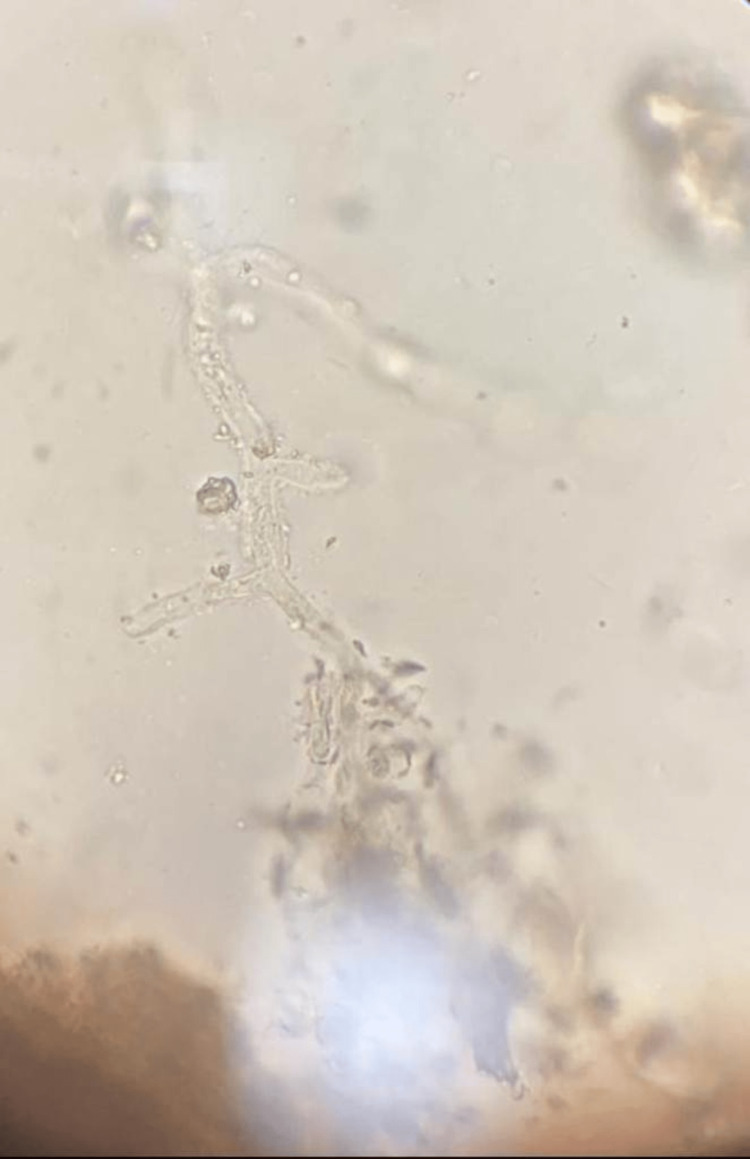
KOH mount picture of third case - mandible mucormycosis

**Figure 6:**
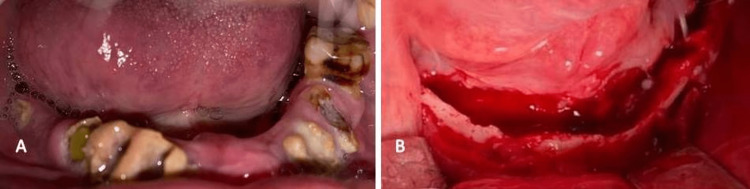
Case three - mandibular mucormycosis (A) Preoperative picture showing poor oral hygiene and pus discharge from mandibular alveolus and (B) intra-operative picture showing debrided mandibular alveolus

A 70-year-old man reported the primary complaint of loosening teeth for the past eight months. The patient had a history of COVID infection, for which he was on steroidal therapy for 12 days. The patient had a history of tooth mobility, six months before acquiring covid infection, and had undergone dental extractions. After the COVID-19 illness, the patient had symptoms of a foul smell, loosening of existing teeth, and pus discharge. On examination, necrotic bone was exposed into the oral cavity with mucosal retraction in the first and fourth quadrants. The dentition was compromised in hygiene and a weak immune barrier system had led to the dissemination of fungi to the mandible and dental extraction before active covid infection may have synergistically helped with fungal growth. The patient was effectively managed by surgical debridement with saucerization and preservation of cortical plates. Infrastructure maxillectomy was done immediately, which helped in further prevention of propagation to the zygomatic-orbital complex. The mandible was treated in the same manner as the above two cases, with the removal of necrotic alveolar bone and preservation of the viable basal bone. The patient received IV amphotericin B for a period and dosage similar to that of the other two patients. There was no residual infection in the one-year follow-up (Figures 5-6).

## Discussion

Mucormycosis is a life-threatening fungal disease present in immune-compromised hosts and is associated with certain independent risk factors like diabetes mellitus (53.6%), long-term steroid therapy, chronic kidney disease, neutropenia, ketoacidosis, etc. [1]. The incidence of mucormycosis in immune-competent patients is rare; however, in recent times, COVID-19 infection has led to immune deregulation, making them susceptible. Overall, the incidence of mucormycosis varies from 0.005 to 1.7/million people and is found to be 80 times higher in India. This makes it the first in most recorded cases, surpassing underdeveloped territories to harbor such an angio-invasive fungal infection [4].

The patients reported here have a common history of past infection with the COVID-19 virus. These cases surged especially after the second wave in India and cannot be precisely ascribed to a particular variant.

The SARS-CoV-2 virus impairs cell-mediated immunity due to a decrease in CD8+ and CD4+ cells, thereby increasing the vulnerability to fungal infections [5]. Patients with an underlying chronic disease or those having high-risk factors along with a new COVID-19 infection are more likely to contract the secondary fungal infection.

Clinically, mucormycosis occurs in one of four forms: rhino-cerebral, pulmonary, gastrointestinal, and disseminated. The rhino-cerebral form is the most common representing form further subdivided into two subtypes: a highly fatal rhino-orbital-cerebral form, which is invasive and may involve the ophthalmic and internal carotid arteries, and a less fatal rhino-maxillary form, which involves the sphenopalatine and greater palatine arteries, resulting in thrombosis of the turbinates and necrosis of the palate [6-8]. Mandibular forms are not included in the classification.

For those COVID-19 patients who had no associated risk factors, the fungal infection could be attributed to the following causes: immune system deregulation, COVID-19-associated coagulopathy [9], vascular invasion by fungal hyphae (raised level of heat shock protein receptor GRP78 on endothelial cells due to stress, which interacts with fungal coat protein CotH3, facilitating fungal endocytosis) [10], long-term unregulated steroid use, free radical-induced endothelial damage, and fungal proliferation as a result of raised serum iron [11]. Any of these factors can help fungal spores germinate and invade the maxillofacial skeleton depending on the progression rate into one of the above clinical forms [12].

Among the reported cases, mandibular mucormycosis is relatively rare, and there are only a few cases of post-COVID mucormycosis of the mandible reported in the literature to date.

Brown and Finn reported a case of mucormycosis of the mandible long back in 1986 in a 57-year-old black man with diabetes mellitus, chronic renal failure requiring dialysis, hypertension, and other medical problems. A total mandibulectomy was performed, sparing the mandibular condyles, with wide local soft tissue debridement along with Amphotericin B therapy [13].

Oswal et al. described a 68-year-old female patient with a significant and long-standing history of diabetes mellitus, hypertension, ischaemic heart disease, diabetic nephropathy, and sleep apnea syndrome who developed fungal osteomyelitis of the posterior mandible. The patient was treated with local necrotic tissue debridement and Amphotericin B therapy, but the patient suffered a cardiac arrest on the eighth post-op day and succumbed to septicemia with multi-organ failure [14].

Dasukil et al. reported three cases of COVID-associated mucormycosis of the mandible, which was successfully treated by surgical debridement and Amphotericin B therapy [15]. The inoculation of spores into the mandibular complex may occur. Our patients had fungal spore dissemination to the mandible, which is a rare phenomenon. Usually, the mandibular apparatus is entirely covered with gingival epithelium in healthy patients, providing a firm barrier to such infections. Mucosal microtrauma may be caused by the humidifiers or associated with independent risk factors like a history of tobacco chewing, poor oral hygiene, and generalized gingival recession as a part of periodontitis. The fungal spores from the sino-maxillary area may breach the periosteum of the lower dentate complex, which is compromised due to the risk factors enumerated above, making the patient vulnerable to developing an extensive disseminated infection of the mandible.

Mortality rates can reach up to 100%, depending on the underlying condition and form of mucormycosis. Early diagnosis, along with treatment of the underlying medical condition, surgical debridement, and administration of antifungals like Amphotericin B intravenously, are required for favorable outcomes [16].

Medical management includes antifungal therapy with Amphotericin B and Posaconazole, along with surgical debridement of necrotic tissue. Surgical debridement or radical resection reduces the fungal overload and thereby prevents progression into sepsis and reduces fatal outcomes.

## Conclusions

Post-COVID mucormycosis is very aggressive, and early diagnosis can result in a reduction in morbidity and functional impairment. Correction of underlying predisposing factors, coupled with an aggressive multi-modality treatment, including surgical debridement and antifungal therapy, remains the cornerstone of the management. The incidence of post-COVID mucormycosis of the mandible is rare, and it deems timely efforts on the part of clinicians to vigilantly identify fungal aetiologies considering the fatality rate.
